# Association between plasma L-carnitine levels and mitochondrial DNA copy number

**DOI:** 10.1186/s12860-023-00496-z

**Published:** 2023-12-11

**Authors:** Mingyue Li, Keming Yang, Immaculata De Vivo, A. Heather Eliassen, Abrar A. Qureshi, Hongmei Nan, Jiali Han

**Affiliations:** 1 Department of Epidemiology, Richard M. Fairbanks School of Public Health, Indiana University, 1050 Wishard Boulevard, RG 6124, Indianapolis, IN 46202-2872 USA; 2 Department of Medicine, University of Pittsburgh Medical Center, Pittsburgh, PA USA; 3 Clinical and Translational Epidemiology Unit, Department of Medicine, Massachusetts General Hospital, Harvard Medical School, Boston, MA USA; 4 Department of Medicine, Harvard Medical School, Boston, MA USA; 5 Channing Division of Network Medicine, Brigham and Women’s Hospital, Boston, MA USA; 6 Department of Epidemiology, Harvard School of Public Health, Boston, MA USA; 7 Program in Genetic Epidemiology and Statistical Genetics, Harvard School of Public Health, Boston, MA USA; 8 Department of Nutrition, Harvard School of Public Health, Boston, MA USA; 9 Warren Alpert Medical School, Brown University, Providence, RI USA; 10 Department of Global Health, Richard M. Fairbanks School of Public Health, Indiana University, Indianapolis, IN USA; 11 Indiana University Melvin and Bren Simon Comprehensive Cancer Center, Indianapolis, IN USA

**Keywords:** Metabolites, L-carnitine, Mitochondria, Mitochondrial DNA copy number, Cross-sectional study, Body mass index

## Abstract

Mitochondria are key cytoplasmic organelles in eukaryotic cells that generate adenosine triphosphate (ATP) through the electron transport chain and oxidative phosphorylation. Mitochondrial DNA (mtDNA) copy number (mtDNAcn) is considered a biomarker for both mitochondrial quantity and function as well as cellular oxidative stress level. Previous epidemiologic findings revealed that weight gain, higher body mass index (BMI), smoking, and high insulinemic potential of lifestyle were associated with lower leukocyte mtDNAcn. Carnitines are a group of compounds that play a critical role in energy production. We quantified the associations of plasma L-carnitine levels with leukocyte mtDNAcn. We then examined the association between mtDNAcn and L-carnitine (HMDB0000062) in 538 U.S. men without cancers, diabetes, or cardiovascular disease at blood collection from the Health Professionals Follow-Up Study (HPFS). We found a significant inverse association between L-carnitine and mtDNAcn (ρ = −0.1, *P* = 0.02). This implies that the carnitine metabolic pathway may be associated with mitochondrial function and oxidative stress.

## Introduction

Mitochondria are multifunctional organelles found in the cytoplasm of eukaryotic cells that play a variety of roles in cellular functions such as energy metabolism, intracellular calcium homeostasis, cell proliferation, and apoptosis [1–3]. The mitochondria produce the vast majority of the energy-rich molecule adenosine triphosphate (ATP) in eukaryotes that do not rely on photosynthesis [2]. Mitochondria, not unexpectedly, are crucial to human health [3–5], and damage to mitochondria is the root cause of many fatal conditions that are inherited matrilineally [5, 6]. Furthermore, mitochondria are profoundly involved in apoptosis and aging [7]. Central to mitochondrial function is mitochondrial DNA (mtDNA), a double-stranded circular DNA molecule, inherited maternally. Comprising approximately 16,569 bp, it encodes a mere 37 genes but is indispensable for mitochondrial operation and ensures nuclear genome stability [8–12].

One molecule in crucial to mitochondrial function is carnitine [13]. It plays a pivotal role in energy metabolism, particularly in tissues such as the heart and skeletal muscles that rely on fatty acid oxidation [14, 15]. Furthermore, carnitine aids in enhancing glucose consumption and maintains its energy-producing capacity despite changes in osmolytic pressure [14, 16–18]. L-carnitine biosynthesis begins with the synthesis of trimethyllysine, then trimethyllysine undergoes enzymatic reactions [17–19]. Notably, L-carnitine has also been shown to have anti-inflammatory and antioxidant characteristics, as well as the capability to improve insulin sensitivity, support protein metabolism, maintain membrane integrity, and address dyslipidemia [17, 18, 20, 21]. Its metabolic role becomes evident when acylcarnitines accumulate due to fatty acid oxidation (FAO), outpacing the tricarboxylic acid cycle (TCA) and affecting insulin sensitivity [18, 20]. By moving acyl CoA derivatives and/or their metabolites out of the mitochondria, L-carnitine can limit their accumulation [17–19]. Consequently, carnitine may serve as an adjunct in the treatment or prevention of insulin resistance and type 2 diabetes [17, 18, 20].

The regulation of mitochondrial DNA copy number (mtDNAcn) is altered in several human mtDNA mutation disorders and is also crucial to a variety of normal physiological processes [22, 23]. Low disease severity and/or inadequate disease penetrance are both associated with high mtDNA copy number in mitochondrial disorders [23]. However, there has been very little research into the link between carnitine metabolites and mtDNAcn. In this study we aimed to examine the association between mtDNAcn and L-carnitine (HMDB0000062) in 538 U.S. men without cancers, diabetes, or cardiovascular disease at blood collection from the Health Professionals Follow-Up Study (HPFS). We found a significant inverse association between L-carnitine (HMDB0000062) levels and mtDNAcn (ρ = −0.1, *P* = 0.02).

## Methods

### Study populations

We used data from the Health Professionals Follow-Up Study (HPFS), a long-time prospective cohort study in the U.S. Details of the HPFS have been previously described. Briefly, the study started in 1986 with 50,529 male health professionals aged 40–75 years [24]. Questionnaires have been mailed to the participants every two years to collect data on lifestyle behaviors, including smoking, physical activity, diet, disease status, and medical history. Blood samples were provided by 18,225 HPFS participants during 1993–1995 [25].

Dietary information and mtDNAcn were measured through blood collection [26]. In the current analysis, we used data from our previous colorectal cancer (CRC) case-control studies nested within the HPFS [27]. A total of 538 male patients from the HPFS with available mtDNAcn were included in the final analysis, which included both CRC cases and controls. The study protocol was approved by the institutional review boards of Brigham and Women’s Hospital and the Harvard T.H. Chan School of Public Health.

### mtDNAcn assessment

The detailed process of mtDNA copy number ascertainment and validation has been published by Meng [26, 28]. To measure the mtDNAcn, total DNA was isolated from buffy-coat fractions using the QIAmp (Qiagen, Chatsworth, CA) 96-spin blood procedure, and pico-green quantification was used to measure the DNA concentrations using a Molecular Devices 96-well spectrophotometer [28]. The DNA concentration was set at 5 ng/μL [28], then the relative mtDNA copy number was determined in a high-throughput 384-well configuration by Applied Biosystems 7900HT Real-Time PCR [28]. Each qPCR experiment utilized 10ng of genomic DNA. We used a multiplex reaction with primers for both the nuclear element AluYb8 and the mitochondrial gene ND2. Specifically, the ND2-forward primer (5’-tgttggttatacccttcccgtacta-3’), ND2-reverse primer (5’-cctgcaaagatggtagagtagatga-3’), AluYb8-forward primer (5’-cttgcagtgagccgagatt − 3’), and AluYb8-reverse primer (5’- gagacggagtctcgctctgtc − 3’) were utilized [28]. The ratio of mitochondrial ND2 gene copy number to genomic single-copy gene copy (N/S) is related to the average mtDNAcn [28]. To assess inter-assay variability, the 10 ng DNA standard curve point in each 384-well plate was employed as calibrator DNA [28]. The relative N/S ratio was computed by subtracting the calibrator DNA’s N/S ratio from each sample’s N/S, which was computed by subtracting the average AluYb8 Ct from the average ND2 Ct value [28].

### Carnitine profiling

Each case-control pair’s samples were sent together, handled the same way, and analyzed in the same run by the same technicians in random order [29–31]. Liquid chromatography tandem mass spectrometry (LC-MS) techniques were used to obtain profiles of plasma L-carnitine [29–31]. Chromatographic retention periods, MS multiple reaction-monitoring transitions, declustering potentials, and collision energies were calculated using reference standards of each metabolite for polar metabolite profiling [30]. Dr. Clary Clish supervised the MIT lab where this scan was performed [32]. He has been studying species identification for over ten years [32].

### Covariate assessment

We included all covariates in the regression model in our study to adjust covariates, including age, race, body mass index, physical activity, smoking status, alternate Healthy Eating Index, alcohol consumption, history of diabetes, history of cardiovascular diseases, and history of hypercholesterolemia, as shown in Table 1. We collected height and weight at baseline, updated weight in a biennial questionnaire, and then calculated BMI using height at baseline and weight at blood sampling. We calculated total activity by summing each activity’s metabolic equivalent hours per week [33].

**Table 1:** Age-standardized characteristics of participants at blood collection according to quartiles of mtDNAcn in overall samples from HPFS

	Overall (N = 538)			
Characteristics	Q1	Q2	Q3	Q4
Number of participants	134	135	135	134
mtDNAcn, z score	-1.2 (0.4)	-0.3 (0.1)	0.3 (0.1)	1.1 (0.3)
Age, years*	67.1 (7.6)	66.2 (7.7)	64.3 (8.2)	64.1 (8.6)
Race, white, %	97.1	93.8	88.4	96.6
Body mass index, kg/m2	26.1 (1.9)	25.8 (2)	26.4 (2.3)	25.6 (2.1)
Physical activity, MET-hours/week	28.5 (16.4)	30.7 (18.8)	29.8 (20.2)	33.1 (20.4)
Smoking status				
Never, %	35.7	44.3	61.8	43
Past, %	52.5	49.4	34.5	53.2
Current, %	11.8	6.2	3.6	3.8
Alternative Healthy Eating Index	47.3 (6.6)	49.3 (8)	51.4 (8.9)	49.6 (7.5)
Alcohol consumption, g/d	15.1 (11.2)	12.7 (11.1)	10.8 (10.1)	14 (11)
History of diabetes	6.2	4.9	1.2	2.4
History of cardiovascular diseases	8.3	7.4	7.4	8.6
History of hypercholesterolemia	38.7	38.1	41.8	39.7

Note: Values are means (SD) for continuous variables, and percentages for categorical variables are standardized to the age distribution of the study population. Overall samples include both CRC cases and controls

* Value is not age-adjusted

### Statistical analysis

We used the server of the Channing Division of Network Medicine at Brigham and Women’s Hospital and Harvard Medical School to calculate age-standard characteristics of participants at blood collection according to quartiles of mtDNAcn in overall samples [34]. We calculated mean and standard deviation for continuous variables and percentages for categorical variables.

We performed age-adjusted Spearman correlation analyses to examine the correlation between carnitine HMDB0000062 and mtDNAcn in overall and control-only samples separately.

We used multiple linear regression models to calculate the mean difference and 95% CI of mtDNAcn across quartiles of carnitine HMDB0000062 in overall and control samples separately. One model is age-adjusted, while the other is multivariate adjusted (MV-adjusted) for age at blood collection, fasting status, case-control status, body mass index (continuous), physical activity (continuous), smoking status (never, former, or current smokers), alcohol consumption (continuous), and Alternative Healthy Eating Index. The significance of the interaction terms was subsequently evaluated using Wald testing. We also drew the figures of the mean difference and 95% CI of mtDNAcn across quartiles of carnitine HMDB0000062 in overall and control samples separately.

We also examined the associations between L-carnitine (HMDB0000062) and BMI, and between BMI and mtDNAcn, also using the age-adjusted model and the MV-adjusted model, described above. All statistical analyses were performed utilizing SAS 9.4 (SAS Institute, Cary, NC). *P* values less than 0.05 on both sides were set to indicate statistical significance.

## Results

We present the age-standardized basic characteristics of our 538 participants at blood collection according to quartiles of mtDNAcn in Table 1. Briefly, the mean ± SD age was 65.4 ± 8.0 y; higher mtDNAcn were associated with younger age. As for race, a higher percentage of white participants was associated with lower quartiles of mtDNAcn (Q1-Q3), and the percentage of white participants was higher in Q4 than in Q3 or Q2. Lower mtDNAcn was associated with higher current smoking rates. Higher physical activity levels and higher Alternative Healthy Eating Index were associated with higher mtDNAcn quartiles, whereas greater alcohol consumption, history of diabetes, and history of cardiovascular disease were associated with lower mtDNAcn quartiles.

Age-adjusted Spearman correlation analyses were performed to examine the relationship between L-carnitine (HMDB0000062) and mtDNAcn, shown in Table 2. A significant inverse relationship between L-carnitine (HMDB0000062) and mtDNAcn was found in both overall samples (ρ = −0.1, *P* = 0.02) and control samples (ρ = −0.15, *P* = 0.01). Data from CRC patients was not included due to concerns about the potential confounding effects of colorectal cancer on the associations we investigated.

**Table 2:** Age-adjusted Spearman correlation between carnitine HMDB0000062 and mtDNAcn in study patients

Cohort	No. of participants	Correlation coefficient	P-correlation
Overall	538	-0.1	0.02
Control	280	-0.15	0.01

Note: overall samples include both CRC cases and controls; and controls include only samples without CRC. P-correlation is *p*-value for correlation

**Fig. 1:**
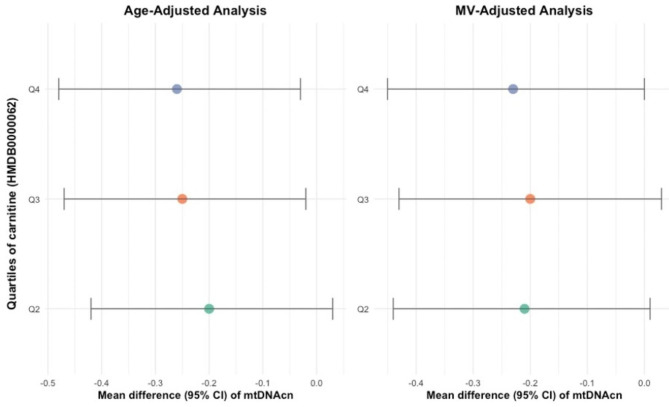
Mean difference (95% CI) of mtDNAcn across quartiles of carnitine (HMDB0000062) in overall study patients

**Fig. 2:**
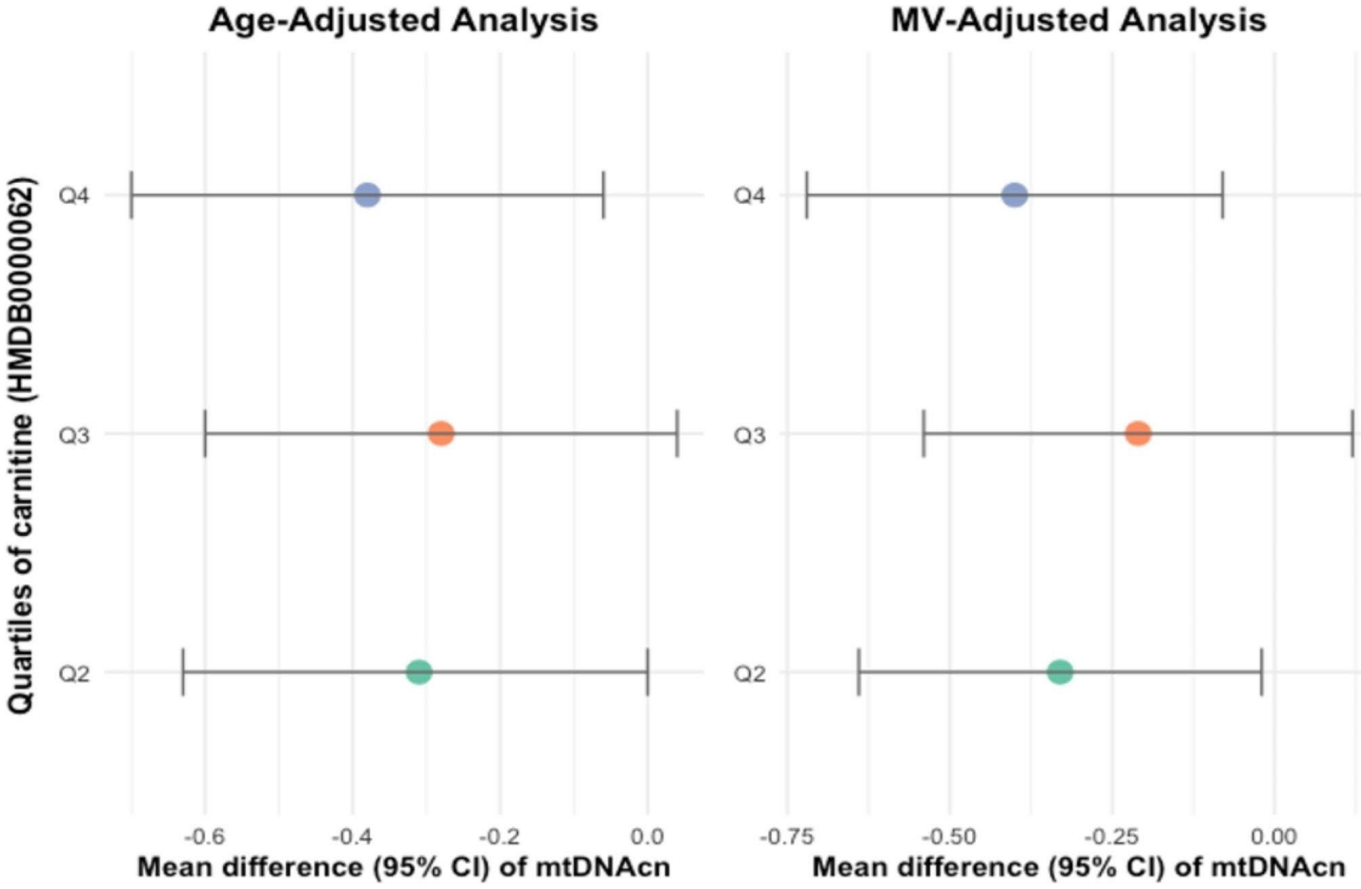
Mean difference (95% CI) of mtDNAcn across quartiles of carnitine (HMDB0000062) in control study patients

The association between L-carnitine (HMDB0000062) and mtDNAcn was further examined using multiple linear regression models (age-adjusted model, and MV-adjusted model, see see Table 3; Figs. 1 and 2). A significant inverse association was found between HMDB0000062 and mtDNAcn in both groups (overall and control samples). In the multivariable-adjusted model, absolute least squares means ± SDs of mtDNAcn across HMDB0000062 quartiles in overall samples were as follows: Q1: reference; Q2: − 0.21 (-0.44, 0.01); Q3: − 0.20 (-0.43, 0.03); Q4: − 0.23 (-0.45, − 0.00); highest Q4 compared with lowest Q1, P-trend = 0.06. Means ± SDs in control samples were as follows: Q1: reference; Q2: − 0.33 (-0.64, − 0.02); Q3: − 0.21 (-0.54, 0.12); Q4: − 0.40 (-0.72, − 0.08); highest Q4 compared with lowest Q1, P-trend = 0.03.

**Table 3:** Mean difference (95% CI) of mtDNAcn across quartiles of carnitine (HMDB0000062) in study patients

	N	Q1	Q2	Q3	Q4	Nominal P for trend
Overall						
Age-adj model	538	ref	− 0.20 (-0.42, 0.03)	− 0.25 (-0.47, − 0.02)	− 0.26 (-0.48, − 0.03)	0.02
MV-adj model	538	ref	− 0.21 (-0.44, 0.01)	− 0.20 (-0.43, 0.03)	− 0.23 (-0.45, − 0.00)	0.06
Control						
Age-adj model	280	ref	− 0.31 (-0.63, 0.00)	− 0.28 (-0.60, 0.04)	− 0.38 (-0.70, − 0.06)	0.02
MV-adj model	280	ref	− 0.33 (-0.64, − 0.02)	− 0.21 (-0.54, 0.12)	− 0.40 (-0.72, − 0.08)	0.03

Notes: covariates adjusted in MV model include age at blood collection, fasting status, case-control status, body mass index (continuous), physical activity (continuous), smoking status (never, former, or current smokers), alcohol consumption (continuous), Alternate Health Eating Index (continuous), and histories of diabetes, cardiovascular diseases, and hypercholesterolemia. P is for *p*-value

There was no significant interaction between L-carnitine (HMDB0000062) and age in either group in the age interaction analysis (Table 4). Some positive relationships were found between L-carnitine (HMDB0000062) and mtDNAcn in some subgroups, including age greater than 67 years in Q3 in both groups (overall: ρ = 0.04, control: ρ = 0.3).

**Table 4:** Mean difference (95% CI) of mtDNAcn across quartiles of carnitine (HMDB0000062) in study patients, stratified by age

	N	Q1	Q2	Q3	Q4	P_1_	P interaction
Overall							
MV-adj model	538	ref	− 0.21(-0.44, 0.01)	− 0.20(-0.43, 0.03)	− 0.23(-0.45, − 0.00)	0.06	
Age = < median (67 yrs)	269	ref	− 0.26(-0.59, 0.08)	− 0.39(-0.72, − 0.06)	− 0.33(-0.66, − 0.003)	0.02	0.08
Age > median (67 yrs)	269	ref	− 0.13(-0.44, 0.17)	0.04(-0.28, 0.37)	− 0.16(-0.49, 0.17)	0.73	
Control							
MV-adj model	280	ref	− 0.33(-0.64, − 0.02)	− 0.21(-0.54, 0.12)	− 0.40(-0.72, − 0.08)	0.03	
Age = < median (67 yrs)	138	ref	− 0.39(-0.87, 0.09)	− 0.55(-1.0, − 0.08)	− 0.48(-0.99, 0.03)	0.03	0.12
Age > median (67 yrs)	142	ref	− 0.21(-0.62, 0.20)	0.30(-0.17, 0.76)	− 0.34(-0.75, 0.07)	0.32	

Notes: covariates adjusted in MV model include age at blood collection, fasting status, case-control status, body mass index (continuous), physical activity (continuous), smoking status (never, former, or current smokers), alcohol consumption (continuous), Alternate Health Eating Index (continuous), and histories of diabetes, cardiovascular diseases, and hypercholesterolemia. P_1_ is for normal *p*-value for trend. P interaction is *p*-value for age interaction

In overall samples, a non-significant positive association between L-carnitine (HMDB0000062) and BMI was found in both models; ρ = 0.01, *P* = 0.35 (ρ = 0.04, *P* = 0.66 in BMI ≥ 25) in the age-adjusted model, ρ = 0.01, *P* = 0.42 (ρ = 0.02, *P* = 0.86 in BMI ≥ 25) in the MV-adjusted model. In control samples, a non-significant positive association between L-carnitine (HMDB0000062) and BMI was also found in both models; ρ = 0.01, *P* = 0.66 (ρ = 0.01, *P* = 0.99 in BMI ≥ 25) in the age-adjusted model, ρ = 0.01, *P* = 0.69 (ρ = 0.02, *P* = 0.42 in BMI ≥ 25) in the MV-adjusted model. In overall samples, a non-significant inverse association between mtDNAcn and BMI was found in both models; ρ = -0.01, *P* = 0.39 (ρ = -0.12, *P* = 0.17 in BM ≥ 25) in the age-adjusted model, ρ = -0.01, *P* = 0.80 (ρ = -0.06, *P* = 0.49 in BMI ≥ 25) in the MV-adjusted model. In control samples, a non-significant inverse association between mtDNAcn and BMI was also found in both models; ρ = -0.02, *P* = 0.38 (ρ = -0.13, *P* = 0.28 in BMI ≥ 25) in the age-adjusted model, ρ = -0.01, *P* = 0.61 (ρ = -0.08, *P* = 0.53 in BMI ≥ 25) in the MV-adjusted model.

## Discussion

In this cross-sectional study, we found that higher levels of carnitine HMDB0000062 are associated with lower mtDNAcn (Table 2). To the best of our knowledge, this is the first epidemiologic study of the association between L-carnitine (HMDB0000062) and mtDNAcn [18]. L-carnitine (HMDB0000062) was positively correlated with BMI (ρ = 0.01, *P* = 0.42 in the MV-adjusted model). This is consistent with previous HPFS studies showing that some carnitine metabolites were positively correlated with BMI [29]. L-carnitines play an important role in mitochondrial long chain fatty acid transport and are essential for maintaining normal mitochondrial activity [17, 20]. Fatty acid oxidation deficiencies may lead to acylcarnitine buildup in people who are overweight and insulin resistant [35, 36]. L-carnitine has also been shown to have anti-inflammatory and antioxidant properties, as well as the capability to improve insulin sensitivity, protein nutrition, dyslipidemia, and membrane integrity [16, 18]. The rate of fatty acid oxidation (FAO) exceeds that of the tricarboxylic acid cycle (TCA), resulting in the buildup of intermediary metabolites including acylcarnitines, which may alter insulin sensitivity [18]. By moving acyl CoA derivatives and/or their metabolites out of the mitochondria, carnitine can limit their accumulation [17, 20]. Consequently, carnitine may serve as an adjunct in the treatment or prevention of insulin resistance and type 2 diabetes [18]. In our study, there was a non-significant inverse association between mtDNAcn and BMI (ρ = -0.01, *P* = 0.80 in the MV-adjusted model in overall samples), which was consistent with earlier findings that lower mtDNAcn was associated with higher BMI [37]. The regulation of mtDNA is altered in several human mtDNA-mutation disorders and is also crucial in a variety of normal physiological processes [1, 22]. Low disease severity and/or inadequate disease penetrance are both associated with high mtDNAcn in mitochondrial disorders [6], and lower metabolic plasticity corresponds with the observed decrease in mtDNAcn [38]. Obesity-related low-grade inflammation decreases mitochondrial energy generation and consumption, which has been proven to exacerbate obesity and create a positive feedback loop that promotes fat storage and weight gain [39].

Therefore, our study suggests that BMI may help explain the fact that mtDNAcn decreased across the increasing quartiles of carnitine HMDB0000062 (Table 3). There could be several reasons for the inverse association between L-carnitine and mtDNAcn. One explanation is that the increased L-carnitine levels in those with greater BMI might be a compensatory mechanism to counteract the impaired mitochondrial function seen in this population. Considering such mitochondrial impairment, increased amounts of L-carnitine may be required to aid in the transport of fatty acids into the mitochondria for energy generation. While those with obesity may have higher L-carnitine levels, their mtDNAcn levels are lower, suggesting that they may still be suffering from mitochondrial dysfunction, which can contribute to a host of metabolic disorders linked to obesity, including insulin resistance and type 2 diabetes. The inverse relationship between L-carnitine and mtDNAcn may also be mediated by other factors related to BMI. For example, chronic inflammation and oxidative stress, which are commonly observed in individuals with obesity, can impair mitochondrial function and reduce mtDNAcn. In this scenario, higher levels of L-carnitine may be a marker of increased oxidative stress and inflammation, which could contribute to the lower levels of mtDNAcn in individuals with higher BMI. Moreover, we found only a modest association between L-carnitine HMDB0000062 and mtDNAcn (Table 2), possibly because of multiple risk factors. Prior epidemiological research demonstrated that weight, the insulinemic potential of lifestyle, BMI, and smoking were inversely associated with mtDNAcn, but whole fruit intake was positively associated with mtDNAcn [26]. The current study contributes to the field by highlighting that high plasma L-carnitine (HMDB0000062) is a possible risk factor for mitochondrial dysfunction.

Interestingly, here the inverse association between L-carnitine HMDB0000062 and mtDNAcn among those aged ≤ 67 y was not detected among some subgroups aged > 67 y (Table 4). Research has shown that aging is inversely associated with mtDNAcn [40]. Accordingly, we would expect to see a more significantly inverse association between carnitine and mtDNAcn. However, the interaction between age and carnitine was not significant in our stratified analysis (Table 4), so this finding in our stratified study may have occurred by chance.

Our study has several strengths. First, the sample size is relatively large, which means it can provide more precise estimates of the association between L-carnitine and mtDNAcn than a smaller study and supports the external validity of our findings. According to our best understanding, both our prior and present studies are the only epidemiologic investigations of the factors that influence mtDNAcn in individuals who are healthy and free of major chronic diseases including diabetes, cardiovascular disease, and cancer. Our findings will facilitate further assessments of mtDNAcn as a possible biomarker for diabetes and other chronic disorders. Moreover, we collected extensive data on covariates and used a systematic method to account for possible batch effects. Furthermore, considering the public health perspective, it is important to understand the how mtDNAcn and L-carnitine contribute to the likelihood of developing diabetes. Early identification, preventative tactics, and targeted therapies can help lessen the impact of diabetes on the population if mtDNAcn and L-carnitine are together validated as a reliable biomarker. These advances can improve quality of life and lead to more cost-effective healthcare strategies, underlining the importance of our research in shaping future public health research.

Nevertheless, our study also has some limitations. First, because our study was cross-sectional, we cannot draw any firm conclusions about causation; more prospective studies are needed to determine whether higher carnitine HMDB0000062 is associated with lower mtDNAcn. Second, our study participants were all from the HPFS, which only included men, so we did not consider any gender influence. Further studies of the gender effect on the association between carnitine HMDB0000062 and mtDNAcn are needed.

In conclusion, our study indicates that higher plasma L-carnitine is associated with lower mtDNAcn. To further verify our findings, more prospective and interventional investigations are necessary.

## Data Availability

The data used in this study are accessible upon reasonable request. Additional information, including procedures for obtaining and accessing the Health Professionals Follow-up Study data, is available at https://sites.sph.harvard.edu/hpfs/for-collaborators/.
